# Case of Laceration of the Perineum

**Published:** 1827-08

**Authors:** C. Williams

**Affiliations:** Surgeon to the Retreat, York.


Case of *Laceration of the Perineum.* By C. Williams, Surgeon to the Retreat, York.

Early in the evening of the 1st of first month, 1825, I was summoned to attend A. W—, æt. twenty-three, in her first labour. The pains were then strong, but soon decreased both in force and frequency. At ten o'clock I gave her a dose of laudanum, and left her. At three the following morning I was again called, when the os uteri was fully dilated, and the pains were strong and forcing; the head continued to advance slowly till nine o'clock, when it pressed firmly on the perineum at each pain, and, as far as I could judge, would have required a dozen more such to dilate the os externum sufficiently to have allowed the head to pass; when, as my fingers were applied to the vertex to prevent its too rapid advancement during a pain, which was not nearly so strong as many that had preceded it, the head suddenly escaped into my hand. I suspected what had taken place, and, after the expulsion of the placenta, I examined the parts, and found that the whole of the perineum and integuments were lacerated to the very centre of the anus, with the exception of the sphincter ani; the fibres of which were distinctly seen through the lacerated opening in the integuments. I informed the patient, as well as her friends, that the parts had been torn during the labour, and that it was necessary to secure them in contact by suture, which was readily agreed to. After washing the parts quite clean, I united the torn edges by three distinct sutures, over which a compress of lint merely was applied. The woman's thighs were kept together by means of a handkerchief tied round them above the knee; and I desired her to lie quite still on her side, and frequently to sponge the part with warm water, especially after any evacuation; and directed a small dose of castor-oil to be taken every morning.

On the fourth day, I examined the parts, and found them looking well. On the ninth, I removed two of the sutures; and, on the twelfth, the remaining one, when the union was complete. A

slight ridge remained in the line of the rent, owing to the thinness of the integuments rendering it necessary to introduce the needle some distance from the edges of the wound. After the last suture was removed, the patient never felt any more uneasiness in the part.

*York; 18th fifth Month*, 1827.

